# Effects of Ambient Ozone Exposure on Mail Carriers’ Peak Expiratory Flow Rates

**DOI:** 10.1289/ehp.7636

**Published:** 2005-03-14

**Authors:** Chang-Chuan Chan, Tsung-Huan Wu

**Affiliations:** Institute of Occupational Medicine and Industrial Hygiene, College of Public Health, National Taiwan University, Taipei, Taiwan

**Keywords:** deviation, lung function, mail carrier, ozone exposure, peak expiratory flow rate

## Abstract

The extent to which occupational exposure to ozone in ambient air can affect lung function remains unclear. We conducted a panel study in 43 mail carriers by measuring their peak expiratory flow rates (PEFRs) twice daily for 6 weeks in 2001. The daily exposure of each mail carrier to O_3_, particulate matter < 10 μm in aerodynamic diameter (PM_10_), and nitrogen dioxide was estimated by one air monitoring station in the center of the mail carrier’s delivery area. Hourly concentrations of air pollutants during their exposure periods were 6–96 ppb for O_3_, 11–249 μg/m^3^ for PM_10_, and 14–92 ppb for NO_2_. Linear mixed-effects models were used to estimate the association between air pollution exposures and PEFR after adjusting for subject’s sex, age, and disease status and for temperature and humidity. We found that night PEFR and the deviation in night PEFR were significantly decreased in association with 8-hr O_3_ exposures with a lag 0–2 days and by daily maximum O_3_ exposures with a lag of 0–1 day in our multipollutant models. By contrast, neither PM_10_ nor NO_2_ was associated with a PEFR reduction. Daily 8-hr mean concentrations of O_3_ had greater reduction effects on PEFR than did daily maximum concentrations. For a 10-ppb increase in the 8-hr average O_3_ concentration, the night PEFR was decreased by 0.54% for a 0-day lag, 0.69% for a 1-day lag, and 0.52% for a 2-day lag. We found that an acute lung function reduction occurs in mail carriers exposed to O_3_ concentrations below current ambient air quality standards and occupational exposure limits.

Epidemiologic evidence suggests that exposures to short-term ambient ozone are associated with consistent and reversible decrements in lung function among children (Burnett et al. 2001; Chen et al. 1999; Hoppe et al. 2003; Jalaludin et al. 2000), the elderly (Hoppe et al. 1995, 2003), and people with a history of respiratory diseases (Hoppe et al. 1995, 2003; Jorres et al. 1996; Kehrl et al. 1999). Recent studies also found that exposures to O_3_ are related to healthy adults’ decreases in lung function, such as forced expiratory volume in 1 sec (FEV_1_), forced vital capacity (FVC), and peak expiratory flow rate (PEFR) (Kinney and Lippmann 2000; Korrick et al. 1998; Naeher et al. 1999; Spektor et al. 1988). These effects usually occur at ambient O_3_ concentrations between 30 and 80 ppb during high O_3_ hours between 0900 and 1700 hr. Such O_3_ concentrations are lower than the U.S. ambient air quality standards for O_3_, which are an 8-hr average at 80 ppb and a 1-hr maximum at 120 ppb, and below the permissible exposure level for workers promulgated by the U.S. Occupational Safety and Health Administration (2004), which is an 8-hr time-weighted average of 100 ppb. Incidentally, the exposure duration between 0900 and 1700 hr described in previous studies happens to be the time when most mail carriers travel door to door to deliver mail and packages in Taiwan. Daytime ambient O_3_ concentrations these mail carriers experience, therefore, are expected to be very close to their occupational exposures. Because potential health effects due to this particular exposure scenario have not been reported before, we conducted this study to assess whether exposure to O_3_ at concentrations below current permissible levels will reduce mail carriers’ lung function.

## Materials and Methods

### Study population.

The study group consisted of 43 mail carriers who were randomly selected from 215 full-time mail carriers working in a main post office of Taichung City, Taiwan. To cover a service area of approximately 10 km^2^ and a half million residents, these mail carriers use either motorcycles or bicycles to deliver mail from 0900 to 1700 hr daily on preassigned delivery routes. A face-to-face questionnaire survey was performed in advance in September 2001 to obtain data from each mail carrier, including age; height; weight; smoking status; disease history of doctor-diagnosed asthma, bronchitis, and pneumonia; and incense burning and environmental tobacco smoke (ETS) exposures at home. Our field study took place from 14 November to 31 December 2001. The Institutional Review Board of National Taiwan University College of Public Health approved the research protocol, and written informed consent was obtained from each participant.

### Lung function measurement.

We chose PEFR as the outcome variable for lung function because it is highly correlated with FEV_1_ in clinical diagnosis (Nowak et al. 1982) and widely used in epidemiology studies (Jalaludin et al. 2000; Krzyzanowski et al. 1992; Naeher et al. 1999; Peters et al. 1999). Each mail carrier was provided with a Midget peak expiratory flow meter (Medget Quan-ding Inc., Taipei City, Taiwan) to measure morning PEFR after awakening and night PEFR between 1000 and 1200 hr daily. Each mail carrier was trained to take three consecutive PEFR readings in the standing position in each measurement. The PEFR measurement was considered valid when the variation of three consecutive readings was < 10%. The best value of three readings was selected for use in further analysis. Our PEFR measurements were conducted between 14 November and 31 December 2001. The PEFR data of the first 3 days were used solely to validate our study subjects’ PEFR measuring technique and were not used in further data analyses. A daily maximum PEFR and daily deviation of PEFR for both morning and night PEFR data were used as outcome variables in our statistical models. Daily deviation of PEFR was defined as the difference between the daily highest PEFR reading and the 6-week average PEFR calculated according to the methods of Pope and Dockery (1992). We present here only the findings of night PEFR to keep our results as concise and informative as possible.

### Monitoring of ambient air pollutants.

**Figure 1:**
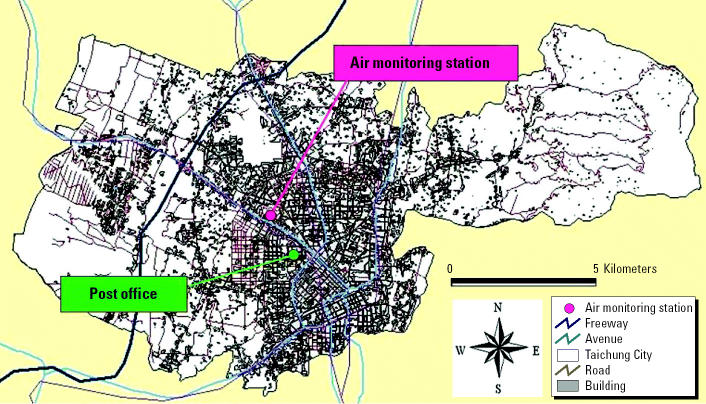
Map of Taichung City.

To estimate the daily exposure of each mail carrier to air pollutants, we abstracted hourly air pollution levels of O_3_, particulate matter < 10 μm in aerodynamic diameter (PM_10_), and nitrogen dioxide from one air monitoring station in the center of each mail carrier’s delivery area according to their daily working hours. The air monitoring station operated in Taichung City, Taiwan, by the Taiwan Environmental Protection Administration (2005) also provided hourly meteorologic data on wind direction, wind speed, temperature, dew point, and precipitation. The locations of the air monitoring station and post office in this study are shown in Figure 1. The environmental data were not used in further data analyses if there were > 20% of hourly values missing in a single day. The 8-hr average and maximum values for O_3_, NO_2_, and PM_10_ between 0900 and 1700 hr were calculated from the data obtained from this monitoring station to represent each subject’s daily exposures to air pollutants. We also summarized meteorologic variables of temperature and relative humidity for the same time segments.

### Statistical methods.

We used a two-step statistical model to estimate the association between PEFR and O_3_ exposures. Multiple linear regressions (MLR) without air pollutants were first used to screen key PEFR-related personal covariates with a *p*-value < 0.25 for further analyses according to the methods of Peters et al. (1999) and Krzyzanowski et al. (1992). In the second step, linear mixed-effects models were used to estimate the pollution effects on PEFR adjusting for personal and meteorologic variables. Such mixed-effects models have the advantage of adjusting for invariant variables by fixed-effects models and accounting for individual differences by random-effects models. We treated subject’s sex; age; body mass index; history of diagnosed respiratory disease; smoking status; air pollutants O_3_, PM_10_, and NO_2_; ambient temperature; and relative humidity as fixed effects and each subject as a random effect in the mixed-effects models. Each of the three air pollutants considered was first put into the linear mixed-effects models separately as single-pollutant models. All of the three pollutants were then jointly put into the linear mixed-effects models as multipollutant models. Air pollution levels with 0- to 3-day lags were used to estimate the time course of pollution effects. Statistical analyses were performed using general additive procedures in the S-PLUS 2000 program (MathSoft Inc., Cambridge, MA, USA). Model selection was based on minimizing Akaike’s information criterion (Akaike 1974).

## Results

### Study population.

As shown in Table 1, there were 39 (91%) males and 4 females (9%) among the 43 mail carriers who participated in the study. The average age was 39 years, and employment duration averaged 13 years. Fifteen (35%) male mail carriers were current smokers. Only a few subjects had a history of doctor-diagnosed respiratory diseases, such as asthma (*n* = 0), bronchitis (*n* = 2), and pneumonia (*n* = 1). Among 43 mail carriers, 15 (35%) were exposed to incense-burning smoke and 9 (21%) were exposed to ETS in their homes.

### Levels of ambient air pollutants and meteorologic parameters.

Table 2 summarizes the O_3_, PM_10_, and NO_2_ concentrations, temperature, and relative humidity. The 8-hr average concentrations (mean ± SD) of air pollutants during the study period were 36 ± 12 ppb for O_3_, 75 ± 38 μg/m^3^ for PM_10_, and 30 ± 10 ppb for NO_2_. The meteorologic conditions were generally mild during the study period with an 8-hr daytime temperature of 19 ± 3°C and a relative humidity of 72 ± 7%. Hourly concentrations of air pollutants in the exposure period were 6–96 ppb for O_3_, 11–249 μg/m^3^ for PM_10_, and 14–92 ppb for NO_2_ during mail carriers’ exposure periods. Pearson correlation coefficients among air pollutants and meteorologic parameters are shown in Table 3. The O_3_ level was not significantly correlated with the other two pollutants, but PM_10_ was highly correlated with NO_2_ (*r* = 0.85). Temperature was also moderately correlated with relative humidity (*r* = 0.46).

### PEFR parameters, and O_3_.

We included sex, age, disease history, temperature, relative humidity and smoking status in the mixed-effects models because our MLR models without air pollutants found that these covariates were associated with PEFR. By contrast, covariates of incense burning and ETS were not included in our second-step models because they were not significantly associated with PEFR. Table 4 lists the results of single-pollutant mixed-effects models separately for O_3_, PM_10_, and NO_2_. Only O_3_ was consistently associated with decreases in night PEFR and the deviation in night PEFR among these three air pollutants. The night PEFR of the mail carriers was significantly reduced in association with 8-hr average O_3_ concentrations with 0- to 2-day lags and maximum O_3_ concentrations during exposure periods with 0- to 1-day lags. The deviation in night PEFR was reduced in association with both 8-hr and maximum O_3_ concentrations with 0- to 2-day lags. Instead of consistent correlation between O_3_ and PEFR, we found NO_2_ effects on both night PEFR and night PEFR deviation at the 2-day lag only, and no PM_10_ effects on either night PEFR or night PEFR deviation.

**Figure 2:**
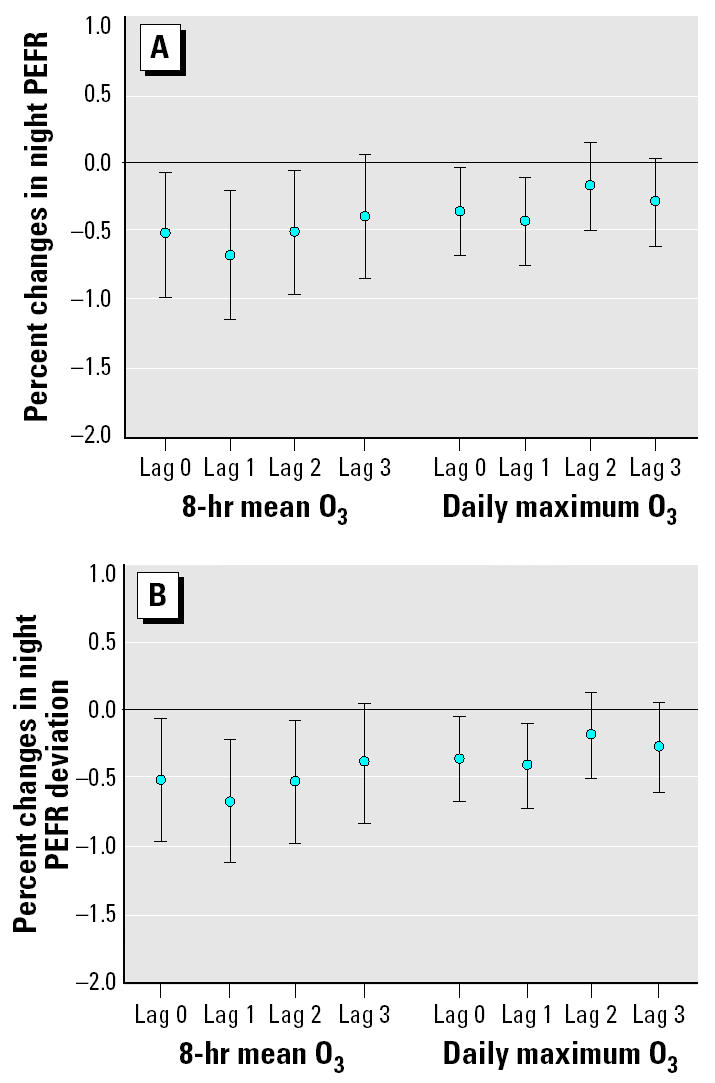
Percent changes in night PEFR (*A*) and night PEFR deviation (*B*) per 10 ppb for 8-hr O_3_ and maximum O_3_. Error bars indicate mean ± SD.

We then put O_3_, PM_10_, and NO_2_ with 0- to 3-day lags in the multipollutant mixed-effects models to estimate the pollution effects on decrease in PEFR by adjusting co-pollutants and key meteorologic factors. We found that O_3_ was associated with PEFR after adjusting for PM_10_, NO_2_, and other covariates. By contrast, PEFR reduction was not associated with either PM_10_ or NO_2_ in the multipollutant models. As shown in Figure 2A, night PEFR and deviation in night PEFR were significantly decreased by O_3_ exposures up to a 2-day lag after adjusting for co-pollutants and key personal covariates. Night PEFR was decreased by 0.54% for 0-day lag, 0.69% for 1-day lag, and 0.52% for 2-day lag. Compared with 8-hr O_3_, 1-hr O_3_ had comparatively less effect on decreasing night PEFR, which was 0.36% for 0-day lag and 0.44% for 1-day lag. As shown in Figure 2B, the effect of O_3_ exposure on the deviation in night PEFR had the same time course as its effects on night PEFR. However, the effects of O_3_ exposure on the deviation in night PEFR were smaller compared with its effects on night PEFR for the same time lag. Our multipollutant mixed-effects models thus showed that ambient 8-hr O_3_ concentrations had greater and longer effects on decreasing PEFR than did maximum O_3_ concentrations during exposure periods. No other covariate except ambient temperature was significantly related to night PEFR and the deviation in night PEFR in our multipollutant mixed-effects models. In addition, subjects’ disease history, including asthma, bronchitis, and pneumonia, had a negative but statistically insignificant influence on PEFR in our multipollutant mixed-effects models. We also found similar O_3_ effects on morning PEFR deviation but not morning PEFR in our multipollutant mixed-effects models (data not shown).

## Discussion

This is the first study to demonstrate that there are effects of occupational O_3_ exposures lagged 0–2 days on reducing mail carriers’ lung function. Such effects can be detected by using either PEFR or PEFR deviation as an indicator of lung function. After occupational exposures during daytime work, night PEFR measurements seem to be more sensitive to O_3_ exposures than are morning PEFR measurements. Because none of our study subject’s daily O_3_ exposure exceeded the hourly standard of 120 ppb, our study supports previous findings from studies in the United States and Canada of a dose–response relationship between lung function change and O_3_ exposure at relatively low daytime ambient concentrations for healthy adults. Exercising healthy adults in New York City (USA) who were exposed to < 80 ppb O_3_ were reported to have a 0.55-L/min decrease in their PEFR per 1 ppb O_3_ (Spektor et al. 1988); healthy women exposed to 8-hr O_3_ at 54 ppb in Connecticut and Virginia (USA) were reported to have a 0.083-L/min/ppb decrease in their PEFR per 1 ppb O_3_ (Naeher et al. 1999); farm workers in Fraser Valley (Canada) who were exposed to a 1-hr daily maximum O_3_ of 40 ppb were reported to have 3.3-mL and 4.7-mL decreases in their FEV_1.0_ and FVC, respectively, per 1 ppb O_3_ (Brauer et al. 1996). A similar dose–response relationship between O_3_ and PEFR reduction was also reported in some European studies. Male cyclists in the Netherlands who were exposed to < 60 ppb O_3_ were reported to have 0.57-L/min decreases in PEFR per 1 ppb O_3_ (Brunekreef et al. 1994); healthy workers and athletes in Germany who were exposed to < 80 ppb O_3_ were also reported to have decrements in their FEV_1_ (Hoppe et al. 1995). Our study also further confirmed that time-weighted O_3_ exposures had greater effects on decreasing lung function than did daily peak concentrations as reported in previous studies (Castillejos et al. 1992; Jalaludin et al. 2000).

Several limitations in our study should be noted. First, the personal O_3_ exposures of mail carriers were not directly measured in this study but were represented by ambient monitoring data. However, the use of fixed-site monitoring data to represent personal O_3_ exposures was not expected to bias our results because the delivery areas of each mail carrier were located within 5 km of the fixed-site monitoring station in this study, and previous studies have shown relatively high spatial representativeness of ambient O_3_ measurements in similar urban environments (Chan and Hwang 1996; Romieu et al. 1998). The lack of personal exposure data could misclassify mail carriers’ actual O_3_ exposures. It has been reported that exposures misclassification can produce biases in both directions for outcomes with multiple risk factors and where exposures are correlated (Zeger et al. 2000; Zeka and Schwartz 2004). Therefore, we cannot entirely rule out the effects of PM_10_ and NO_2_ on reducing mail carriers’ PEFR in this study. PM_10_ does not distribute throughout an air shed as thoroughly as O_3_, and its use may have introduced more exposure misclassification for that pollutant. This may partially explain the lack of an observed effect on PEFR by relatively high acute PM_10_ exposures in this study. Another potential confounding factor of our findings was that some unmeasured air pollutants, such PM_2.5_ and volatile organic compounds from tailpipe emissions, could also have been responsible for lowering lung function rather than O_3_ alone in our study.

Despite these limitations, our data generally support the finding that a lung function reduction occurred among mail carriers exposed to daily O_3_ concentrations below current ambient air quality standards and occupational exposure limits. O_3_ is a strong oxidant that can induce pulmonary function impairment at low levels via several toxicologic mechanisms. For example, O_3_ can trigger the neutral receptors of the airway by inducing lipid peroxidation and the production of cycloxygenase (Hazucha et al. 1996) or increase respiratory allergy or reduce resistance to respiratory tract infections by suppressing T_H_^1^ cells in the immune system (Van Loveren et al. 1996). More recently, O_3_ exposure was found to induce mild and moderate respiratory response among children in Taipei by causing DNA breaks and impairing pulmonary cells (Cheng et al. 2003). Because O_3_ pollution is still widespread in major metropolitan areas worldwide, more studies are needed to elucidate clinical significance of O_3_ effects on lung function at low exposure levels, especially for susceptible populations.

## Figures and Tables

**Table 1:** Basic characteristics of 43 mail carriers participating in the study (PEFR measurement period from 17 November through 31 December 2001).

Characteristic	Male	Female	Total
No. of subjects (%)	39 (91)	4 (9)	43
Age [years (mean ± SD)]	38.1 ± 9.6	39.7 ± 4.4	39 ± 8
Work [years (mean ± SD)]	12.2 ± 6.7	11.3 ± 0.5	13 ± 6
Height [cm (mean ± SD)]	169.0 ± 4.9	160.4 ± 8.4	167.9 ± 5.5
Weight [kg (mean ± SD)]	66.8 ± 9.6	62.8 ± 5.3	65.8 ± 7.1
Disease history			
Asthma [*n* (%)]	0 (0)	0 (0)	0 (0)
Bronchitis [*n* (%)]	2 (5)	0 (0)	2 (5)
Pneumonia [*n* (%)]	1 (3)	0 (0)	1 (2)
Smoking status			
Current smoker [*n* (%)]	15 (38)	0 (0)	15 (35)
Nonsmoker [*n* (%)]	24 (57)	4 (100)	28 (60)
ETS at home [*n* (%)]	9 (23)	0 (0)	9 (21)
Incense burning at home [*n* (%)]	13 (33)	2 (50)	15 (35)
No. of PEFR measurements	986	87	1,073

**Table 2:** Summarized statistics for air pollutants and meteorologic data during the study period (14 November through 31 December 2001).

Variable	No.	Mean ± SD	Minimum	Maximum
8-hr average during exposure periods*^a^*				
O_3_ (ppb)	44	35.6 ± 12.1	7.6	65.1
PM_10_ (μg/m^3^)	43	74.7 ± 37.9	19.1	213.8
NO_2_ (ppb)	43	30.0 ± 10.1	17.3	65.9
Temperature (°C)	45	19.1 ± 3.4	12.2	24.2
Relative humidity (%)	45	71.5 ± 6.6	59.0	88.0
Maximum during exposure periods				
O_3_ (ppb)	44	52.6 ± 18.8	5.6	95.5
PM_10_ (μg/m^3^)	43	106.8 ± 44.8	11.4	249.0
NO_2_ (ppb)	43	52.9 ± 21.8	14.0	91.6

a Mail carriers’ exposure periods are about 8 hr between 0900 and 1700 hr every working day.

**Table 3:** Pearson correlation coefficients for air pollutants and meteorologic data during the study period (14 November through 31 December 2001).

Pearson correlation coefficients	O_3_	PM_10_	NO_2_	Temperature	Relative humidity
O_3_	1.000				
PM_10_	0.211	1.000			
NO_2_	0.093	0.854**	1.000		
Temperature	0.010	0.402**	0.353*	1.000	
Relative humidity	−0.413**	0.088	−0.063	0.460**	1.000

* *p* < 0.05;

** *p* < 0.01.

**Table 4:** Regression coefficients (95% CIs) of individual pollutants on PEFR estimated by single-pollutant linear mixed-effects models.

	8-hr average for exposure period			Hourly maximum for 8-hr exposure period		
	O_3_	PM_10_	NO_2_	O_3_	PM_10_	NO_2_
Night PEFR						
Lag 0	−0.33* (−0.44 to −0.18)	0.02 (−0.03 to 0.07)	0.09 (−0.06 to 0.23)	−0.20* (−0.26 to −0.08)	−0.01 (−0.03 to 0.06)	−0.01 (−0.09 to 0.05)
Lag 1	−0.38** (−0.50 to −0.22)	0.04 (−0.03 to 0.06)	0.19 (0.04 to 0.34)	−0.22* (−0.26 to −0.08)	0.01 (−0.04 to 0.04)	0.08 (−0.02 to 0.15)
Lag 2	−0.32* (−0.42 to −0.15)	−0.04 (−0.10 to −0.01)	−0.26 (−0.46 to −0.10)	−0.17 (−0.23 to −0.04)	−0.05 (−0.05 to 0.01)	−0.18* (−0.27 to −0.10)
Lag 3	−0.22 (−0.34 to −0.05)	0.02 (−0.01 to 0.07)	0.08 (−0.11 to 0.25)	−0.09 (−0.17 to 0.00)	−0.02 (−0.06 to 0.01)	0.08 (−0.02 to 0.17)
Night PEFR deviation						
Lag 0	−0.32* (−0.43 to −0.18)	−0.00 (−0.04 to 0.04)	0.11 (−0.03 to 0.25)	−0.19* (−0.27 to −0.11)	−0.02 (−0.05 to 0.02)	−0.01 (−0.08 to 0.06)
Lag 1	−0.38** (−0.51 to −0.26)	0.02 (−0.03 to 0.06)	0.17 (0.02 to 0.32)	−0.20* (−0.29 to −0.12)	−0.02 (−0.05 to 0.02)	0.06 (−0.01 to 0.13)
Lag 2	−0.32* (−0.44 to −0.19)	−0.07 (−0.12 to −0.03)	−0.26 (−0.41 to −0.11)	−0.16* (−0.25 to −0.08)	−0.04 (−0.07 to 0.00)	−0.18* (−0.25 to −0.11)
Lag 3	−0.22 (−0.35 to −0.09)	0.01 (−0.04 to 0.05)	0.06 (−0.10 to 0.22)	−0.11 (−0.20 to −0.03)	−0.01 (−0.04 to 0.02)	0.07 (0.00 to 0.15)

* *p* < 0.05;

** *p* < 0.01.
